# γ-Radiation Induces Long-Term Dose-Related Proteomic and Phosphorylated-Tau Species Changes in Non-Human Primate Hippocampus

**DOI:** 10.21203/rs.3.rs-8426282/v1

**Published:** 2026-01-19

**Authors:** Kathleen Hatch, Erin K. Murphy, John D. Olson, Robert N. Cole, Robert N. O’Meally, Roxann G. Ingersoll, Daniel P. Perl, George W. Schaaf, J. Mark Cline, Diego Iacono

**Affiliations:** Uniformed Services University (USU); Uniformed Services University (USU); Wake Forest University School of Medicine; Johns Hopkins University; Johns Hopkins University; Johns Hopkins University; Uniformed Services University (USU); Wake Forest University School of Medicine; Wake Forest University School of Medicine; Uniformed Services University (USU)

**Keywords:** non-human primate brain, γ-radiation, hippocampus radiosensitivity, proteomic changes, apelin receptor, neurological long-term post-radiation effects, Tau-phosphorylation, neurodegeneration markers, cognitive deficits

## Abstract

**Background:**

Long-term neurological consequences of acute total-body γ-radiation are poorly understood. Here we have investigated the persistency of molecular changes in non-human primates (NHPs) more than five years after a single exposure to either a ~ 4Gy or ~ 8Gy dose.

**Methods:**

MS-based proteomic analyses were performed on samples of micro-dissected hippocampus from the brains of irradiated NHPs, having received 4 or 8Gy total-body γ-radiation at least 5 years previously. Interpretation of proteomic data was subsequently expanded through STRING and DAVID analyses. Confirmation of proteomic findings was further assessed using Western blot and digital-PCR. Where applicable, data was analyzed using Student t-tests.

**Results:**

Proteomic analysis of the hippocampus revealed profound, dose-dependent molecular changes. The ~ 4Gy dose primarily impacted synaptic plasticity, while the ~ 8Gy dose affected catabolism, vascular development, and inflammation. Apelin receptor (APLNR) was the only protein consistently lowered at both radiation doses, suggesting it may be a key biomarker for radiation-induced brain injury. Significantly, while most proteins linked to neurodegeneration were unchanged, Tau protein (MAPT) levels increased at the ~ 8Gy dose, and some animals developed Tau-positive lesions resembling neurofibrillary tangles. The study also found complex, dose-specific alterations in phosphorylated forms of other key proteins like alpha-synuclein and TDP-43.

**Conclusion:**

Overall, the findings demonstrate that different radiation doses induce distinct and persistent molecular changes in the NHP hippocampus. This work highlights the dysregulation of proteins associated with neurodegeneration as a major long-term consequence of radiation, providing new insights into the molecular basis for post-exposure cognitive deficits.

## Introduction

Exposure to high-dose γ-radiation can cause severe and life-threatening health consequences, including acute radiation syndrome (ARS), late delayed radiation-induced brain injury (RIBI), and the delayed effects of acute radiation exposure (DEARE), which often manifest as chronic, multi-organ damage^1-3^. To better understand and mitigate these risks, particularly following accidental or intentional radiation events, the use of a translationally relevant animal model is essential. Non-human primates (NHPs) are considered the gold standard for such studies due to their close physiological, immunological, behavioral, and genetic similarities to humans, which allow for more accurate investigations of radiation-induced pathology, and brain pathology in particular^1,4^. Among the organ systems vulnerable to radiation, the central nervous system (CNS) is of particular concern due to its integral role in an organism’s overall health and function. However, the long-term effects, their molecular mechanisms, and late clinical manifestations of sublethal γ-radiation exposures to the brain tissue and function remain poorly understood.

Previous studies in other large animal models, such as swine, have provided some initial insight into the effects of a single acute total-body low-dose (1.79Gy) γ-radiation exposure on various molecules and anatomical regions of the brain^5^. These recent findings in swine revealed that an acute total-body low-dose sublethal radiation exposure can induce complex region-specific and molecular alterations across various regions of the brain, which may be either detrimental or beneficial if related to a specific clinical or occupational context^6-7^. For instance, a surprising reduction in proteins typically associated with neurodegenerative pathologies such as phosphorylated-Tau (pTau) - a hallmark of Alzheimer’s disease (AD) - was observed in the hippocampus of swine^5^; or a reduction of lrrk2 levels in the Striatum of the same irradiated animals (lrrk2 levels are usually increased in patients with a diagnosis of Parkinson’s disease [PD])^6^. Overall, these recent findings in a large animal (swine) highlighted the intricate and hormetic nature of the γ-radiation^8-11^ on the brain both in general, and in specific regions (i.e., hippocampus vs. striatum). These initial findings in large animals (swine) underscored the necessity to conduct similar in-depth, possibly long term, investigations in an even more translationally relevant model such as non-human primates (NHPs).

While several studies of NHPs have utilized histopathological tissue analysis and robust imaging techniques like magnetic resonance imaging (MRI), susceptibility weighted imaging (SWI) and diffuse tensor imaging (DTI), to identify vascular, inflammatory, and white matter related changes in NHP brains after irradiation, there is still much work to be done to reveal, for example, the exact molecular underpinnings of radiation-induced cognitive disfunctions^1^, which affect a consistent number of patients after irradiation for brain tumor treatments^12-13^.

To date, studies that combine specific brain region analysis with powerful molecular techniques like unbiased mass-spectrometry based proteomics validated by orthogonal methodologies like quantitative Western blotting (WB) (with use of total protein levels for normalization procedures), digital PCR (dPCR) measures, and polymer-based automated immunohistochemistry (IHC) methods for irradiated NHPs brains were lacking.

This study aimed to characterize the long-term persistent proteomic changes in the hippocampus of young adult NHPs years after a single, acute, total-body γ-radiation exposure at different doses. The hippocampus was initially selected as the specific brain region of interest due to its critical role in learning, memory, and cognition in general, and its known vulnerability in both neurodegenerative diseases and post-radiotherapy cognitive decline^14-16^. Crucially, this study aimed to address a possible fundamental mechanism in the etiology of dementia: an acute environmental insult, such as ionizing radiation exposure, in initiating long-term neurodegenerative cascades. By characterizing the persistent shift in the proteome of non-human primate brain, and specifically of hippocampus, we specifically aimed to identify molecular pathways - ranging from synaptic dysregulation to the failure of Tau proteostasis (especially pTau-181 and pTau217 abnormalities) - that mirror the pathology of sporadic dementias such as AD. Also, we aimed to provide then direct evidence that γ-radiation exposure at certain doses can act as a prodromal driver for dementia, or a specific type of it, offering so a unique translational model to dissect how proteomic re-arrangement may precipitate pathways linked to specific proteinopathies characteristic of cognitive decline in the context of dementia processes. Specifically, we employed an unbiased mass spectrometry (MS)-based proteomic approach to simultaneously measure thousands of hippocampal proteins, enabling a comprehensive survey of molecular alterations in irradiated (4Gy and 8Gy) vs. control (non-irradiated/0Gy) NHPs in that specific region of the brain. More specifically, our primary goals were:

(1) to identify possible significant and durable proteomic changes in the hippocampus of irradiated vs. control animals;

(2) to determine if these changes linearly correlated with the radiation doses (3.5-4.0Gy [4Gy group] vs. 8.05–8.5 Gy [8Gy group]) (**Supplemental Table T1**);

(3) to assess abnormalities of brain molecular pathways and key markers associated with neurodegenerative disorders such as Alzheimer's disease (AD), Parkinson's disease (PD), and Frontotemporal Dementia (FTD).

Moreover, to validate the proteomic findings, we planned to confirm the key proteomic changes using independent orthogonal methods, including Western blotting (WB) and digital PCR (dPCR), alongside with a systematic neuropathological immunohistochemistry assessment of the hippocampal region comparing irradiated vs. sex- and age-matched control animals. By pursuing these goals, we seek to provide a deeper understanding of the molecular and histopathological long-term consequences of γ-radiation exposure to the brain, and to the hippocampus in particular. Furthermore, it should be noted that the exposure ranges selected here for investigation fall on either side of the LD50.

## Material and Methods

### Brief description of the colony/subjects and animal handling

Subjects in this study were selected from a larger cohort of rhesus macaques (*Macaca mulatta*) known as The Wake Forest University Nonhuman Primate Radiation Late Effects Cohort (NHP RLEC) and housed at the Wake Forest University School of Medicine (WFUSM). Described previously^65^, the cohort is a collection of NHPs accumulated gradually over two decades to facilitate the study of delayed effects of irradiation in a large animal model with a high degree of genetic similarity to humans (~ 93% DNA sequence homology with homo sapiens). Briefly, the RLEC includes approximately 220 living animals (male and female) in total, observed daily and subject to clinical examinations, routine blood and fluid collections, noninvasive imaging including magnetic resonance imaging (MRI), computed tomography (CT), ultrasound (US), and DEXA (dual energy x-ray absorptiometry), as well as approximately 160 deceased animals who, upon reaching humane endpoints, received euthanasia and underwent full necropsy examinations with collection of fixed and frozen tissue and viable cells. A large portion of the RLEC (~ 300 animals) were exposed to irradiation (described in more detail below) at various institutions as part of different acute studies of radiation effects and medical radiation countermeasures, while approximately 80 of the animals were never irradiated and serve as controls. Animals were adopted into the cohort and continue to be adopted as they become available. All procedures were carried out in compliance with the ARRIVE guidelines (https://arriveguidelines.org/arrive-guidelines).

Animals consumed a Western Diet (Typical American Diet; Purina LabDiet 5L0P, Richmond, IN, USA) supplemented with fresh fruits and vegetables and were provided with water *ad libitum*. They were housed socially in indoor-outdoor pens whenever possible (90% of the time), or in group cages if necessary for safe handling or medical care. Care was taken to ensure compatibility amongst grouped animals. Environmental enrichment (including fruits/vegetables, toys, puzzles, climbing and hiding environments) was provided continuously on a rotating basis. Additionally, an independent behavioral management team monitored the animals’ behavioral well-being and made recommendations as needed. All animals were trained to cooperate in handling procedures, to minimize stress. Sampling was scheduled so that the animals were sedated the minimum number of times required for data collection.

For the present study, a total of 14 male NHPs (9 irradiated and 5 non-irradiated controls) were selected from the RLEC who had available brain tissue in the cohort tissue biorepository and received a dose of 3.5-4 Gy irradiation (n = 5) or 8.05–8.5 Gy irradiation (n = 4) or no irradiation (**Supplemental Table T1**). Animals who met these criteria were selected to yield groups similar in age and time since irradiation as possible. A summary of animal features and experimental timepoints is presented in **Supplemental Table T1**. All subjects in this study were male, due to availability.

### Summary of the radiation procedures

The control and irradiated animals used for this study were obtained from: University of Maryland, University of Illinois, Armed Forces Radiobiology Research Institute, Citox Labs, and Primate Products. Irradiated animals received 3.5 to 8.5 Gy total body radiation under IACUC oversight at their prior institution using one of two strategies: (1) linear accelerator-derived photons at a nominal mean energy of 2 MeV, delivered at 80 cGy/minute as a split dose given half anterior-posterior and half posterior-anterior; or (2) Cobalt 60-derived gamma irradiation delivered simultaneously, bilaterally at 60 cGy/min. Note that these are potentially lethal doses: the LD 10/30 for rhesus macaques is ~ 5.5 Gy, the LD 50/30 is ~ 6.7 Gy, and the LD 90/30 is 8 Gy^66^. Depending on the approved protocols of the originating institutions, animals were either awake (chair or standing trained (n = 2)) during irradiation procedures or administered ketamine while breathing room air (no correlation was observed between sedation method and proteomic outliers or radiosensitivity). Surviving animals were subsequently transferred to Wake Forest University School of Medicine for long-term monitoring post-radiation. Irradiation methods, supportive care strategies, and acute effects, where available, have been previously reported^65,67,68^.

### Brief description of clinical and necropsy procedures

After arrival at Wake Forest, all animals were monitored twice daily by trained veterinary and technical laboratory staff to assure animal well-being and social stability. Acute illness and chronic conditions were monitored and classified using a standard nonhuman primate-specific modification of the Children’s Clinical Oncology Group toxicity criteria^69^. Sick animals were promptly evaluated by one of six institutional veterinarians independent of the research team, two of whom are board-certified by the American College of Laboratory Animal Medicine. Animals were euthanized after 1.4–13.7 years of recovery (4 Gy and 8 Gy groups, euthanized when necessary, according to standard criteria) via intravenous pentobarbital (30 mg/kg), a method approved the American Veterinary Medical Association and the Wake Forest University IACUC. Following the pentobarbital injection, full-body tissue perfusion with cold, sterile saline was performed, and brains were removed, sectioned coronally into 4 mm thick slabs, frozen on dry ice, and stored at −80°C until use.

### Tissue homogenization for proteomic and WB procedures

Hippocampi were dissected from 4 mm thick sections of frozen whole brain. These slices were utilized for fresh tissue samples by several investigators, and, in most cases, hippocampus was only available from 1 hemisphere of the brain slice. For proteomic analysis, hippocampal tissue (~ 600–900 mg per animal) was homogenized using glass dounce homogenizers with ice cold lysis buffer (1mL/100 mg tissue) containing: 50mM Tris–HCl (pH = 8), 1% Igepal, 150mM NaCl, 1 mM EDTA, 1mM PMSF, 1mM NaF, 1:100 protease inhibitor cocktail (Sigma-Aldrich, P2714, St. Louis, MO, USA). Following centrifugation for 20min at 12,000g, supernatants were collected and aliquoted for freezing at −80°C. Micro BCA assay (Thermo-Fisher Scientific, cat. #23235, Waltham, MA, USA) was used to determine total protein content.

Samples were subsequently used for MS-based proteomic analysis and Western blot validation.

### Proteomic methods

#### Isobaric Mass Tag Labeling and Fractionation:

Protein extracts were buffer exchanged using SP3 paramagnetic beads (GE Healthcare) (Hughes CS et al, Nature Protocols 2019 14(1):68–85). Briefly, samples were brought up to 100 uL with 10 mM TEAB + 1% SDS and disulfide bonds reduced with 10 uL of 50 mM dithiothreitol for 1 hour at 60C. Samples were cooled to RT and pH adjusted to ~ 7.5, followed by alkylation with 10 uL of 100 mM iodoacetamide in the dark at RT for 15 minutes. Next, 100 ug (2 uL of 50 ug/uL) SP3 beads were added to the samples, followed by 120 uL 100% ethanol. Samples were incubated at RT with shaking for 5 minutes. Following protein binding, beads were washed with 180 uL 80% ethanol three times. Proteins were digested on-bead with trypsin (Pierce) at 37C overnight (5 ug enzyme) and labeled with a unique TMTpro 16-plex reagent (Thermo Fisher, LOT # YK388744) according to the manufacturer’s instructions. All 12 TMT labeled peptide samples were combined, dried by vacuum centrifugation, resuspended in 100 μL 200mM TEAB buffer and filtered through Pierce Detergent removal columns (Fisher Scientific PN 87777) to remove excess TMT label, small molecules and lipids. Peptides in the flow through were diluted to 2 mL in 10 mM TEAB and fractionation on a XBridge C18 Column (5 μm, 2.1 x 100 mm column (Waters) using a 0 to 90% acetonitrile in 10 mM TEAB gradient over 85 min at 250 μL/min on an Agilent 1200 series capillary HPLC with a micro-fraction collector. Eighty-four 250 ul fractions were collected, concatenated into 24 fractions^70^, and dried by vacuum centrifugation.

#### Mass Spectrometry analysis:

Peptides in each of the 24 fractions were analyzed by reverse-phase chromatography tandem mass spectrometry on a Neo Vanquish UPLC interfaced with an Orbitrap Exploris 480 mass spectrometer (Thermo Fisher Scientific). Peptides were introduced onto a 2 cm trapping column before being separated using a 2%–95% acetonitrile in 0.1% formic acid gradient over 90 min at 300 nl/min. The 75 μm x 25 cm column was packed in house with ReproSIL-Pur-120-C18-AQ (2.4μm, 120 Å bulk phase, Dr. Maisch) and ionized at 2200 volts using a Pepsep Emitter setup (Bruker, Billerica MA). MS2 survey scans of precursor ions were acquired in a 3 sec cycle time, from 375–1500 m/z at 120,000 resolution at 200 m/z, automatic gain control (AGC) of 3e6. The RF lens was set to 45% and the internal mass calibration of the instrument was enabled. Precursor ions were individually isolated within 0.7 m/z by data dependent acquisition with a 45s dynamic exclusion and fragmented using an HCD activation with a collision energy of 36. Fragment ions were analyzed at 30,000 resolution and an AGC setting of 1e5 with TurboTMTPro setting enabled.

#### Data analysis:

Fragmentation spectra were processed by Proteome Discoverer v3.1 (PD3.1 ThermoFisher Scientific) and searched with CHIMERYS using inferys 3.0.0 fragmentation model against the Uniprot Macaca mulatta database downloaded on 240624. Search criteria included trypsin as the enzyme, two missed cleavages, and 20 ppm tolerance on fragment ions. TMTpro on N-terminus and K and carbamidomethylation on C were set as fixed modifications and oxidation on M was set to variable. Peptide identifications from the CHYMERYS searches were processed within PD3.1 using Percolator at a 1% False Discovery Rate. Peptide spectral matches (PSMs) were filtered for Isolation Interference with a Normalized CHIMERYS Coefficient of 0.8 Relative protein abundances of identified proteins were determined in PD3.1 from the normalized median ratio of TMT reporter ions, having signal to noise ratios > 4, from all PSMs from the same protein. Technical variation in ratios from our mass spectrometry analysis is less than 10%^71^.

### Western Blot (WB) procedures

Protein samples were mixed with dH2O and 10% by volume 10x NuPAGE Sample Reducing Agent (Life Technologies, cat#2353153, Carlsbad, CA, USA), 25% by volume 4x Laemmli Sample Buffer (Bio-Rad Laboratories, Inc., cat#1610747, Hercules, CA, USA), and a calculated volume of protein to achieve 2μg/μl concentration, denatured for 10 min at 70°C. For all brain regions, 20μg of protein per sample (Control n = 5, 4 Gy n = 5, 8 Gy n = 4) were loaded into Novex Nupage 4–12% Bis-Tris Gels (Life Technologies, cat#NP0329, Carlsbad, CA, USA) for 30min electrophoresis at constant 200V, with 2.5μl Precision Plus Protein Dual Color Standards (Bio-Rad Laboratories, cat#1610374, Hercules, CA, USA) loaded into one well per gel. Gels were transferred using the iBlot2 dry transfer method (Life Technologies, cat#IB21001, Carlsbad, CA, USA) onto PVDF membranes and then rinsed with dH2O and incubated at RT for 10 min with No-Stain Protein Labeling Reagent (Thermo-Fisher, cat#A44449, Waltham, MA, USA) according to manufacturer’s instructions for total protein fluorescent visualization. Membranes were then rinsed with dH2O and blocked for 1h at room temperature in 5% milk in 1x TBST.

Primary antibodies were diluted in 5% milk in 1x TBST to appropriate working concentrations and incubated on the membranes at 4°C overnight. After rinsing the membranes 3x 5min in 1x TBST, they were incubated for 2h at RT in appropriate HRP tagged secondary antibodies diluted 1:2000 in 5% milk in 1x TBST. Membranes were rinsed in 1x TBST for 3x 5min and in 1x TBS for 1x 5min, then incubated for 1 min in chemiluminescent substrate (SuperSignal West Pico Chemiluminescent Substrate, Thermo-Fisher Scientific, cat#34577, Waltham, MA, USA) and imaged on iBright Imaging System (Invitrogen, ThermoFisher Scientific, Waltham, MA, USA).

Densitometry was performed using iBright Analysis Software (Thermo-Fisher Scientific, Waltham, MA, USA), with all protein intensities normalized to total protein signal intensity. All WBs were run in triplicate and averaged for each antibody.

#### Primary antibodies:

anti APLNR (1:500, cat#702069, Invitrogen, Thermo-Fisher Scientific, Waltham, MA, USA), anti CAMKII-delta (CAMK2D) (1:1000, cat# PA5-22168, Invitrogen, Thermo-Fisher Scientific, Waltham, MA, USA), anti CHRM5 (1:500, cat# PA5-106925, Invitrogen, Thermo-Fisher Scientific, Waltham, MA, USA), SST (1:500, cat# 701935, Invitrogen, Thermo-Fisher Scientific, Waltham, MA, USA), anti GFAP (1:20000, cat# NCL-L-GFAP-GA5, Leica, Deer Park, IL, USA), anti AIF1/IBA1 (1:2000, cat# ab178847, abcam, Waltham, MA, USA), anti HT7 (1:40000, cat# MN1000, Invitrogen, Thermo-Fisher Scientific, Waltham, MA, USA), anti pTau-S202 (1:10000, cat# ab108387, abcam, Waltham, MA, USA), anti pTau-S202/T205 (1:500, cat# MN1020, Invitrogen, Thermo-Fisher Scientific, Waltham, MA, USA), anti pTau-T217 (1:1000, cat# 44-744, Invitrogen, Thermo-Fisher Scientific, Waltham, MA, USA), anti pTau-T181 (1:5000, cat# MN1050, Invitrogen, Thermo-Fisher Scientific, Waltham, MA, USA), anti APP (1:2500, cat# MAB348, Millipore Sigma, Burlington, MA, USA), anti α-synuclein (1:20000, cat# ab138501, abcam, Waltham, MA, USA), anti phospho-α-synuclein (1:500, cat# GTX5022, GeneTex, Irvine, CA, USA), anti TDP-43 C-terminal (1:5000, cat# 81350-1-RR, Proteintech, Rosemont, IL, USA), anti TDP-43 N-terminal (1:1000, cat# 10782-2-AP, Proteintech, Rosemont, IL, USA), and anti pTDP-43 (S403/404) (1:1000, cat# 66079-1-Ig, Proteintech, Rosemont, IL, USA).

#### Secondary antibodies:

HRP tagged Goat anti-Mouse (1:2000, ab97040, abcam, Waltham, MA, USA), HRP tagged Goat anti-Rabbit (1:2000, ab97080, abcam, Waltham, MA, USA).

### RNA isolation / cDNA synthesis and digital Polymerase Chain Reaction (dPCR) Procedures

Tissue samples (20 mg per sample) were homogenized using an Omni Bead Ruptor (cat. 19-050A) and added to Zymo 2mm Bead Bashing tubes (cat. S6003-50) containing 350ul of a working solution of Buffer RLT Plus from the RNeasy Plus Mini Kit (Qiagen cat. 74134) and β-ME (Sigma Aldrich cat. 1610710). Parameters for the Bead Ruptor were 4m/s, 45 sec, 1 cycle, 0 dwell. The program was run for 4 cycles with a 30 second hold/dwell on ice in between each cycle. The tubes were then spun at 3000g x 5 minutes and isolated on the Qiagen QIA-cube Connect (cat. 9002864) using the standard RNeasy Plus Mini protocol with the elution volume set to 40ul. RNA samples were quantified using the Qubit RNA Broad Range Assay Kit (Thermofisher cat. Q10210) with the Qubit 4 fluorometer (Thermofisher cat. Q33226), and 390ng total of RNA was converted into cDNA for each RNA sample using the SuperScript VILO cDNA Synthesis Kit (Thermofisher cat. 11754050). The thermocycler conditions were: 25°C x 10 min, 42°C x 60 min, 85°C x 5 min, 4°C hold.

Absolute quantification of cDNA was performed using TaqMan gene expression assays (Thermo-Fisher Scientific, Waltham, MA, USA; *MAPT* (Rhesus macaque, assay ID: Rh04269822_m1 – Cat# 4331182; VIC dye) and *APLNR* (Rhesus macaque, assay ID: Rh03043249_s1 – Cat# 4351372; FAM dye)). The assays were multiplexed and analyzed with digital PCR (dPCR) through the Johns Hopkins Genetic Resources Core Facility (GRCF), SOM, JHU; RRID:SCR_018669. One water control was used with each multiplex on each plate. Viability and proper dilution of the samples for each assay were tested. It was determined that 1:10 dilutions were appropriate for both assays. Samples were run on QIAcuity 8 instrument, firmware version 3.0, analysis software version 3.1.0.0. Each sample was run in triplicate, loading 5 μl into 7.2 μl of mastermix. The 12.2 μl of mastermix was then loaded into the nanoplate, with 8 μl distributed across each well by the instrument, and samples were thermocycled using the standard probe cycling conditions in the QIAcuity user manual: hold 95°C for 120sec, and then 40x cycle of 95°C for 15 sec and 60°C for 30 sec. Differences in RNA expression were evaluated by ordinary one-way ANOVA of “Conc. [cp/ÂμL] (undiluted sample)” values, with statistical significance *p* < 0.05.

### Neuropathology and Immunohistochemistry procedures

Each formalin-fixed hemi-brain was cut into a series of 4–5 mm thick coronal slabs and tissue samples were sub-dissected for processing to paraffin blocks using an automated tissue processor (ASP 6025, Leica Biosystems, Nussloch, Germany). Paraffin embedded tissue blocks were cut in a series of 20, 5-μm thick consecutive sections. The first three sections were selected for hematoxylin and eosin (H&E), while the remaining sections were retained for immunohistochemistry (IHC) procedures. For each antibody, IHC procedures were performed using a Leica Bond III automated immunostainer with a diaminobenzidine chromogen detection system (DS9800, Leica Biosystems, Richmond, IL, USA).

All stained sections were digitally scanned using an Aperio scanner system (Aperio AT2—High Volume, Digital whole slide scanning scanner, Leica Biosystems, Inc., Richmond, IL, USA) and stored in a digital image archive for further assessment and analyses. Preliminary neuropathologic assessments for each section were performed using Aperio ImageScope (Aperio ImageScope, version 2016, Leica Biosystems, Inc., Richmond, IL, USA) to verify presence of immunoreactivity (IR), anatomical localization and neurohistological distribution of each antibody. After the preliminary ImageScope histological inspections (magnification at 20×), a Zeiss Imager A2 (ImagerA2 microscope, Zeiss, Munich, Germany) bright-field microscope with higher magnification lenses (40×, 63×-oil immersion objective) was used to identify and photograph histologic and pathologic details, as needed.

#### IHC antibodies:

anti-phosphorylated tau (pTau) AT8 (1:200, cat# MN1020; Invitrogen, Thermo-Scientific, Waltham, MA); anti-1–42 β-amyloid (1–42 βA) 4G8 (1: 500, cat# SIG-39220; BioLegend, San Diego, CA); anti-amyloid precursor protein APP A4 (1: 100, cat# MAB348; EMD Millipore, Burlington); anti-glial fibrillary acidic protein GFAP (1:1000, cat# NCL-L-GFAP-GA5; Leica Biosystems, Wetzlar, Germany); anti-ionized calcium-binding adapter molecule 1 AIF1/Iba-1 (1: 500, cat# 019-19741; FUJIFILM Wako Pure Chemical Corporation, Osaka, Japan).

### Statistics

For the MS-proteomics data analyses and methods see paragraph Tandem Mass Tag (TMT) proteomics procedures and data analysis. P-values were calculated using t-test for individual proteins with biological replicates (4Gy vs Control, 8 Gy vs Control, and 8Gy vs 4Gy). For grouped protein abundances, coefficients of variation were calculated (CV = 100×std. dev/median) and Z-score transformation of normalized protein abundances from a quantitative proteomics analysis using isobaric mass tags was applied before performing the hierarchical clustering based on Euclidean distance and complete (furthest neighbors) linkage. Densitometry data from WB were obtained using iBright Analysis Software (Thermo-Fisher Scientific, Waltham, MA, USA) and analyzed by 2-tailed, unpaired t-ests using GraphPad Prism v10.5.0 (La Jolla, CA, USA), with a threshold of p < 0.05 used to determine significant differences. All WB experiments were performed in technical triplicate and target/whole protein relative density ratios were averaged between the same samples for analysis. dPCR technical triplicates were averaged for analysis of undiluted sample concentrations between groups with 1-tailed, unpaired t-tests using GraphPad Prism v10.5.0 (La Jolla, CA, USA), with significance threshold set at p ≤ 0.05. Outliers were determined using Grubb’s method, and Bartlett’s test for homogeneity of variance was used to test for significant variability between subjects.

## Results

### MS-based proteomic analysis

Protein abundances in hippocampal protein extracts from 4Gy, 8Gy and Control animals were highly consistent for reliable MS-based proteomic analysis (**Supplemental Fig. F1a**). Unsupervised Principal Component Analysis (PCA) (**Supplemental Fig. F1b**) demonstrated a clear separation between groups and showed 4Gy and 8Gy groups diverging from the Control group, often in opposite directions. Overall, these initial group-related findings suggested that the effects of radiation on proteomic profiles, at least in the hippocampus, do not follow strictly or necessarily a dose-effect linear relationship. Indeed, this variable response across samples was also observed in the protein abundances heat map (**Supplemental Fig. F1c**).

**Figure 1:**
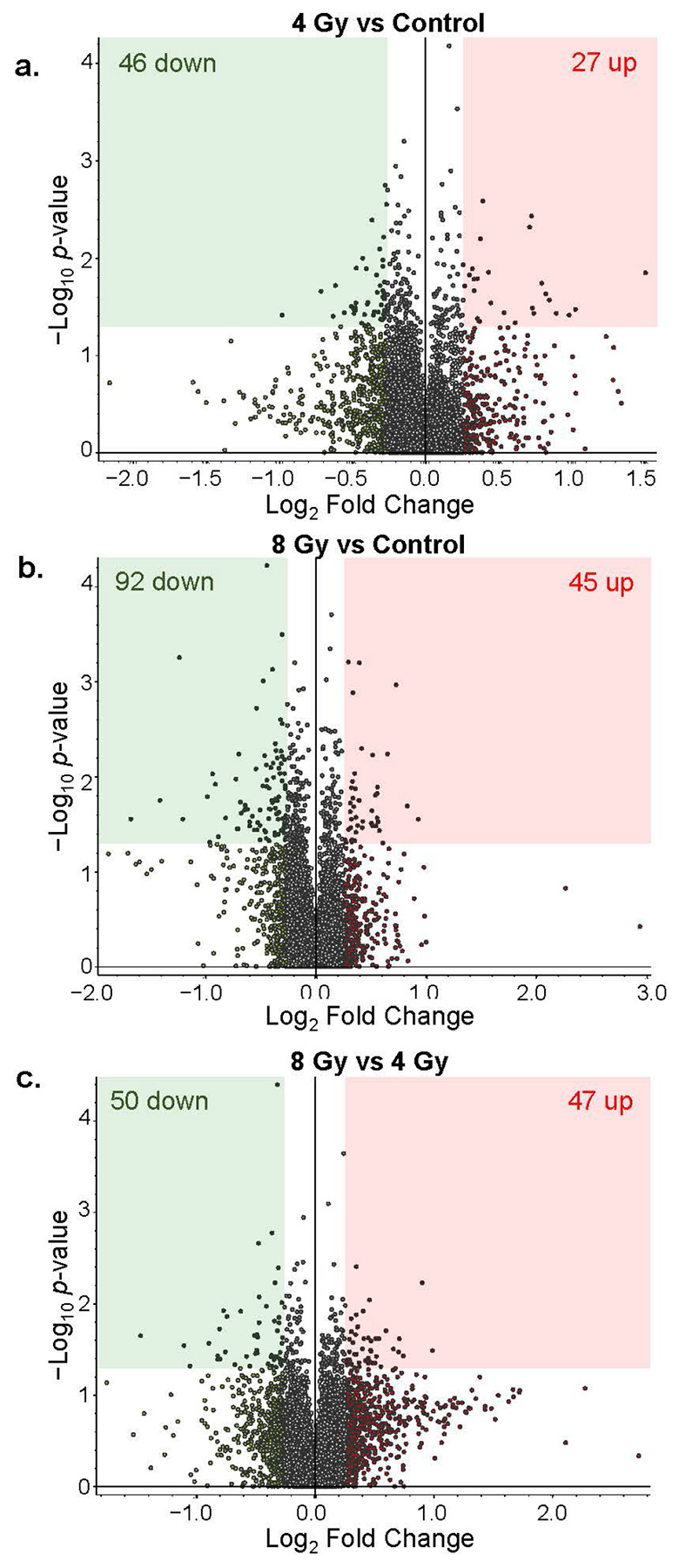
Volcano plots of primate hippocampal proteomic profiles. Log_2_ (Fold Change) ratios plotted against −Log_10_ (p-values) of protein abundances for the comparisons 4 Gy vs Control (a), 8 Gy vs Control (b), and 8 Gy vs 4 Gy (c). Decreased proteins are shown in green, and increased proteins are shown in red, with corresponding shaded areas indicating which proteins fall within our threshold (p<0.05; Log_2_ (FC)=0.26).

**Figure 2:**
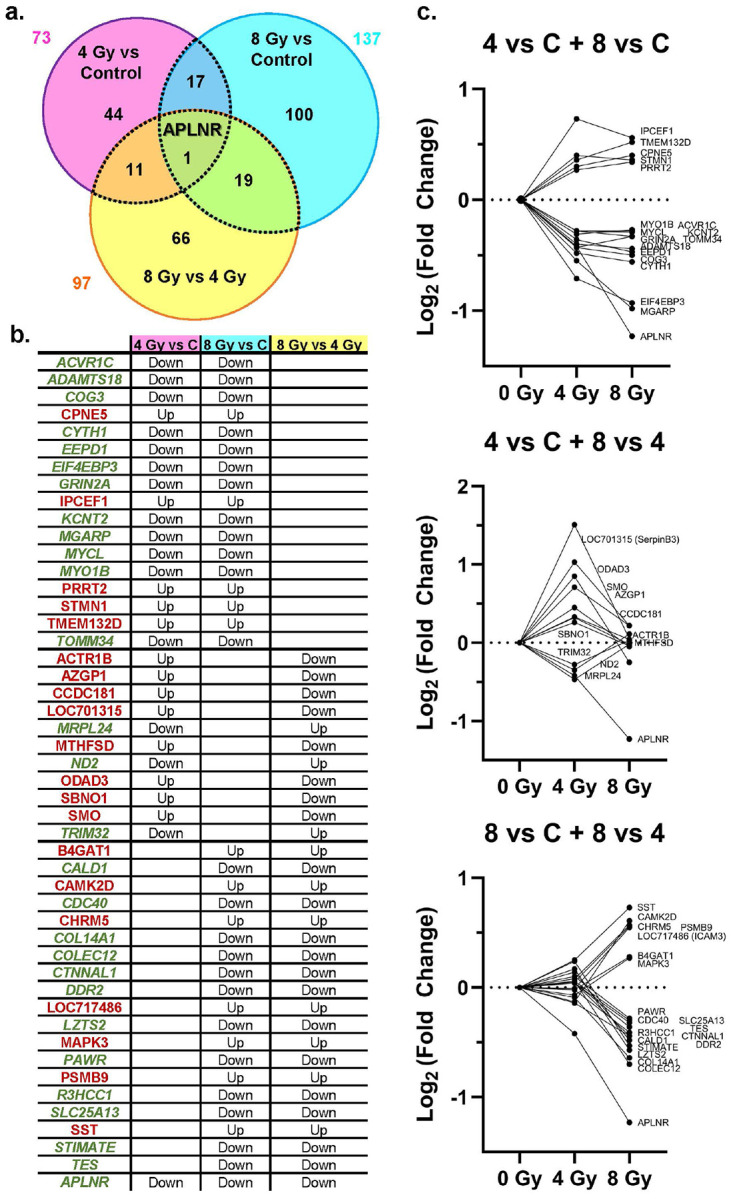
Legend not included with this version.

Through MS-proteomic analysis we identified 12,181 protein groups from 144,594 peptide groups sequenced, with 290,482 peptide-spectral matches at 5% false discovery rate (full list of proteins in Table S1). Comparisons were performed between 4Gy vs. Control, 8Gy vs. Control, and 8Gy vs. 4Gy group. Based on the established thresholding parameters (significance with a *p* < 0.05; Log_2_ Fold Change (FC) = 0.26), we were able to identified 27 proteins with increased (red) abundance and 46 proteins with decreased (green) abundance in the hippocampi of 4Gy vs. Control NHPs (Fig. 1a); 45 increased and 92 decreased proteins in the hippocampi of 8Gy vs. Control NHPs (Fig. 1b); and 47 proteins with increased abundance and 50 proteins with decreased abundance in 8Gy vs. 4Gy NHPs (Fig. 1c). We have plotted the significant proteins for each comparison on the volcano plots of the other comparisons to demonstrate distinct distributions of increased and decreased proteins that change between the groups (**Supplemental Fig. F2**). This exercise helps to identify the most promising proteins of interest (and thus pathways) associated with these two levels of irradiation. In fact, a comparison of significant proteins revealed only moderate overlaps between conditions, with apelin receptor (APLNR) being the only protein consistently significant (lowered levels) across all comparisons (Fig. 2). While APLNR demonstrates an almost linear, dose-dependent response, the other overlapping proteins show an interesting pattern of differential response to radiation (Fig. 2c). Those proteins significant for both the 4 Gy vs C and 8 Gy vs C comparisons show consistent directions of change after both radiation doses, with many of the proteins appearing to potentially reach a threshold level due to irradiation. Significant proteins specifically affected by 4 Gy exposure (where abundance levels after 8 Gy exposure are more similar to those of controls) are highlighted by the overlap of 4 Gy vs C and the 8 Gy vs 4 Gy comparisons. And finally, overlapping significantly altered proteins for both the 8 Gy vs C and 8 Gy vs 4 Gy comparisons identify proteins uniquely affected by 8 Gy, where abundance levels after 8 Gy exposure diverge drastically from the levels seen after 4 Gy exposure. The full lists of proteins identified as significant for each comparison are detailed in **Supplemental Spreadsheet S1**. The TMT-MS raw data and analysis files are uploaded to the publicly accessible repository MassIVE (ftp://massive-ftp.ucsd.edu/v11/MSV000099483/).

### Expanding proteomic findings using STRING and DAVID

**Figure 3:**
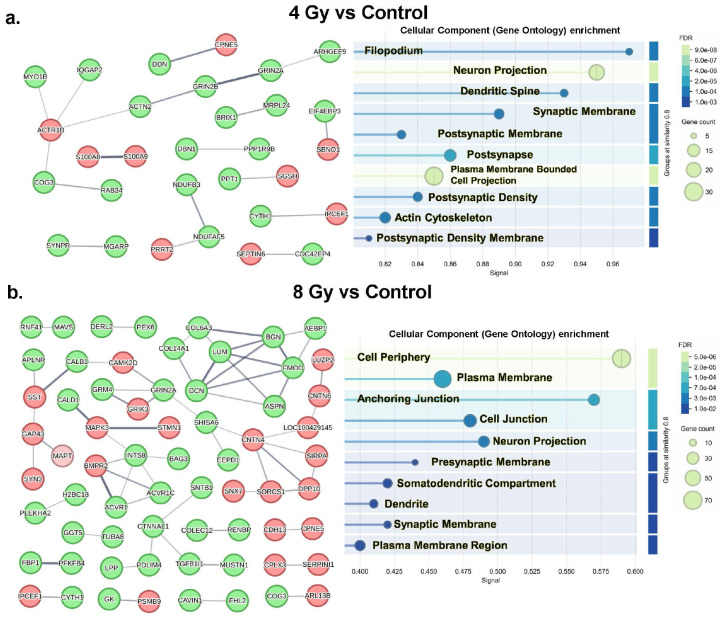
STRING interaction networks of proteins significantly altered between Control, 4 Gy & 8 Gy. Protein-protein network interactions from significantly altered proteins identified by MS-based proteomic analysis comparing 4 Gy vs Control (a) and 8 Gy vs Control (b). Proteins without any interactions were omitted from these displayed networks, although the full networks are displayed in Supplemental Fig. 4 (including the network for 8 Gy vs 4 Gy) and were used for the STRING analyses including Gene Ontology enrichment. MAPT was included in the network analysis but only shows interactions with a single protein (GAP43) in the 8 Gy vs Control comparison. STRING enrichment results showing cellular components implicated by the interaction networks are presented here for comparison. Biological processes and molecular functions (when available) are shown in Supplemental Figure 3.

In an effort to explore the biological implications of the MS-based proteomic findings, we subsequently utilized the STRING (https://string-db.org/) database to perform protein-protein interaction analyses. Briefly, STRING is a powerful tool using data mining to provide network interactions information and visualization based on functional associations and known physical interactions derived from scientific literature and computational predictions^17^. We input all proteins within our threshold parameters for each comparison to generate three distinct STRING interaction networks: 4Gy vs. Control (Fig. 3a, **Supplemental Fig. F3a**), 8Gy vs. Control (Fig. 3b, **Supplemental Fig. F3b**), and 8Gy vs. 4Gy (**Supplemental Fig. F3c**). Interestingly, despite the many proteins entered in the network, there was a large proportion of proteins with very few (if any) interactions. This seemed to suggest that the most prominent effects of radiation engage specific and diverse biological components without necessarily consistently activating the same networks or broad networks. More specifically, based on the STRING enrichment analysis of the 4Gy vs. Control network, there was some preferential effect on biological components related to synaptic plasticity and neurotransmission pathways (Fig. 3a, **Supplemental Fig. F4a**). In contrast, the 8Gy vs. Control network shows an enrichment among proteins predominantly involved in cellular components and collagen binding (Fig. 3b, **Supplemental Fig. F4b**). Curiously, the 8Gy vs. 4Gy network did not show much enrichment of any pathways (**Supplemental Fig. F4c**). Interestingly though, when the significant proteins from all comparisons were input to create an interaction network (**Supplemental Fig. F5a**), we observed a clear prevalent pattern of effect on synaptic transmission/plasticity and structural protein binding emerge (**Supplemental Fig. F5b**). It is also worth noting that when included in the interaction networks, MAPT (Tau protein) only showed a single interaction with GAP43 (synaptogenesis marker) in the 8Gy vs Control comparison (Fig. 3b, **Supplemental Fig. F5a**).

Additionally, we utilized another powerful tool, the Database for Annotation, Visualization and Integrated Discovery (DAVID), which uses a vast knowledge base to derive biological meaning from large gene datasets through functional annotation (https://david.ncifcrf.gov/)^18-19^. For the purposes of this investigation, we input the significantly altered proteins (using official gene symbols) from each comparison condition into DAVID’s functional annotation tool against a *Macaca mulatta* background to generate three separate gene ontology (GO) analyses (**Supplemental Table T2**).

For the 4Gy vs. Control comparison, the DAVID analysis identified 4 significantly enriched (*p* < 0.05) molecular functions (78.4% coverage) (where coverage indicates the proportion of input genes identified/included in the enrichment results), 16 significantly enriched cellular components (94.6% coverage), and 13 significantly enriched biological processes (89.2% coverage). The GO (gene ontology) results indicate an impact of 4Gy γ-radiation vs. Control on glutamate ion channel and receptor activity (molecular function), neuronal and synaptic elements (cellular components), calcium signaling, autophagy, actin filament organization and bundle assembly, and astrocytic and neuronal development (biological processes).

By contrast, for the 8Gy vs Control comparison, the analysis identified 6 significantly enriched molecular functions (91.1% coverage), 15 significantly enriched cellular components (91.1% coverage), and 11 significantly enriched biological processes (91.9% coverage). The GO results indicate an impact of 8Gy radiation on cadherin binding and oxygen sensor activity (molecular function), neuronal and synaptic membrane elements (cellular components), catabolic processes, blood vessel development and regulation of chemotaxis (biological processes).

Finally, for the 8Gy vs. 4Gy comparison, the analysis identified 4 significantly enriched molecular functions (81.4% coverage), 4 significantly enriched cellular components (93.8% coverage), and 2 significantly enriched biological processes (86.6% coverage). Moreover, the GO results indicated a possible differential impact of 8Gy vs. 4Gy γ-radiation on RNA, actin filament and calmodulin binding (molecular function), cytoskeletal elements (cellular components), and negative regulation of fibroblast proliferation (biological processes).

Taken together, these results suggest great diversity in the proteins affected by irradiation, while the molecular mechanisms behind these alterations remain obscured, with few clear pathways implicated by these secondary analyses.

### Western Blot-verified proteomic findings

**Figure 4:**
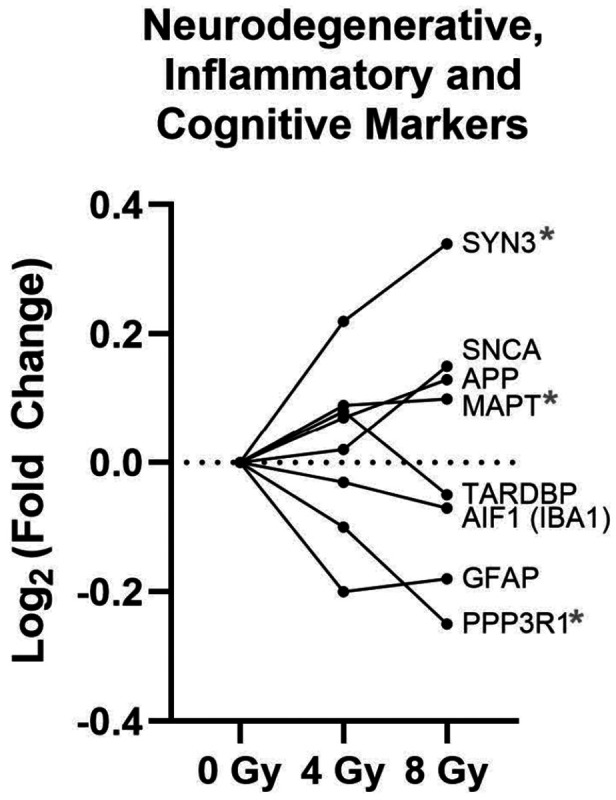
MS-based proteomic measures for select canonical markers involved in neurodegeneration, inflammation and cognition. Changes in relative protein abundance levels (Log_2_(FC) across radiation exposures (4 Gy and 8 Gy) are presented for select neurodegenerative (APP, MAPT, SNCA (α-synuclein), TARDBP (TDP-43)), inflammatory (GFAP, AIF1 (IBA1)), and cognitive (SYN3, PPP3R1) markers. MAPT, SYN3 and PPP3R1 were significantly altered (*p* < 0.05) after 8 Gy exposure (indicated by red *).

In light of the aging phenotype of NHPs at this chronic timepoint, we chose to investigate how different levels of γ-radiation may have impacted the most common neurodegenerative pathways by checking the proteomic data for six proteins typically associated with various neurodegenerative processes (apolipoprotein precursor protein [APP], MAPT [Tau], SNCA [α-synuclein], TARDBP [TDP-43], GFAP and AIF1/IBA1). We determined that of those six targeted molecules, only MAPT (tau protein) showed significant (though subtle) long-term changes in abundance (proteomic data) in the hippocampus after acute total-body γ-radiation (*p*=0.025, Log_2_ (FC)= 0.10). Specifically, an increase in MAPT levels was present in the 8Gy vs. Control group (Fig. 4, Table 1). Then, to determine which specific type of tau (i.e., HT7, pTau-S202, pTau-S202/T205, pTau-181, pTau217) was driving the observed increase, we performed a series of Western Blot (WB) quantifications on the same hippocampal tissue homogenate samples. Through WBs quantifications, we sought to confirm the unaltered abundance of APP, α-synuclein, phospho-α-synuclein, TDP43, phosphor pTDP43, GFAP and AIF1/IBA1 as well.

Furthermore, we selected other four additional molecular targets (APLNR, CAMK2D, CHRM5, SST) to confirm with WB-based quantifications their significantly altered abundances in the 8Gy vs. Control group. The selection of these four additional targets was based on the MS-based proteomic findings that showed APLNR significantly decreased in all three comparisons, with CAMK2D, CHRM5, and SST significantly increased in the 8Gy vs. 4Gy comparison (Fig. 2). These additional targets were selected after narrowing the original threshold criteria to proteins showing greater than 40% change up or down and after finding the proteins as having a significant and directionally consistent change in abundance in at least 2 out of 3 comparisons. The selected targets exhibited some of the most extreme FC values in the 8Gy vs. Control comparison, which we interpreted as increasing the likelihood of congruent verification by WB analyses.

**Figure 5:**
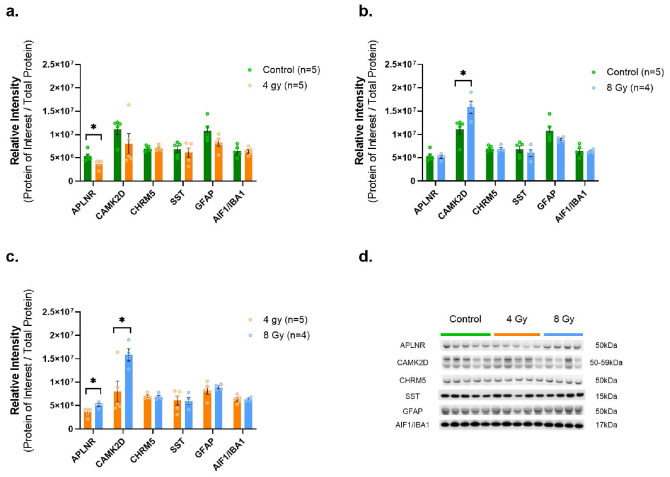
WB verifies abundance levels of select MS-based proteomic markers. Protein levels of Protein levels of APLNR are significantly decreased after 4 Gy exposure (**a.**), and levels of CAMK2D are significantly increased after 8 Gy exposure (**b.**), while CHRM5, SST, GFAP and IBA1/AIF1 remained unchanged (**a.-c.**). (**d.**) Representative western blots for APLNR, CAMK2D, CHRM5, SST, GFAP and IBA1/AIF1 in hippocampus. Each blot was run in triplicate with all protein signal intensities normalized to total protein content. The graphs depict the average of 3 runs. *Indicates p<0.05 as determined by unpaired 2-tailed t-test. Error bars represent the standard error of the mean (SEM). Original full-length blots and total protein stain for each are presented in Supplementary Figure F7.

WB analyses identified and confirmed a significant decrease in APLNR protein levels among animals exposed to 4Gy vs. Control (p = 0.0271, df = 8) (Fig. 5a), while there was no measurable difference for the 8Gy vs. Control group, although there was a significant increase in the 8Gy group vs. 4Gy (p = 0.024, df = 7). We also confirmed a significant increase in CAMK2D protein levels after 8Gy exposure vs. both Control (p = 0.0293, df = 7) (Fig. 5b) and 4Gy (p = 0.0262, df = 7) (Fig. 5c). Moreover, we did not identify significant changes via WBs in CHRM5, SST as well as GFAP and AIF1/IBA1 levels (with these last two results consistent with the proteomic results) for any group comparison.

**Figure 6:**
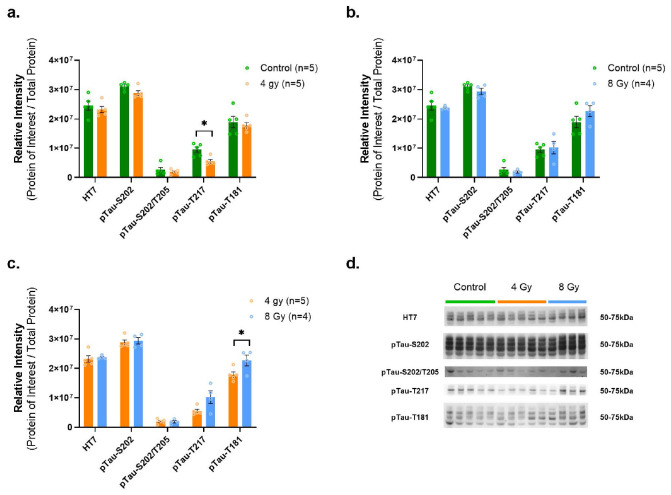
WB determines complex impact of irradiation on long-term abundance levels of select Tau variants. Our analysis of tau proteins revealed decreased pTau-T217 after 4 Gy (**a.**) and a trend towards increased pTau-T181 after 8 Gy exposure (**c.**), but all other measured variants were unchanged (**a.-c.**). (**d.**) Representative western blots for HT7, pTau-S202, pTau-S202/T205, pTau-T217 and pTau-T181 in hippocampus. Each blot was run in triplicate with all protein signal intensities normalized to total protein content. The graphs depict the average of 3 runs. * indicates p<0.05 as determined by unpaired 2-tailed t-test. Error bars represent the standard error of the mean (SEM). Original full-length blots and total protein stain for each are presented in Supplementary Figure F8.

As for MAPT/Tau WB analyses, amongst the different types of Tau measured such as HT7 [total tau] and tau phosphorylated at different sites (pTau-S202, pTau-S202/T205, pTau-T217, pTau-T181), pTau-T217 showed a significant decrease after 4Gy vs. Control group (p = 0.0086, df = 8) (Fig. 6a), while pTau-T181 was significantly elevated in 8Gy vs. 4Gy exposed animals (p = 0.0431, df = 7) (Fig. 6c), with a similar trend (increase) for the 8Gy vs. Control (Fig. 6b).

**Figure 7:**
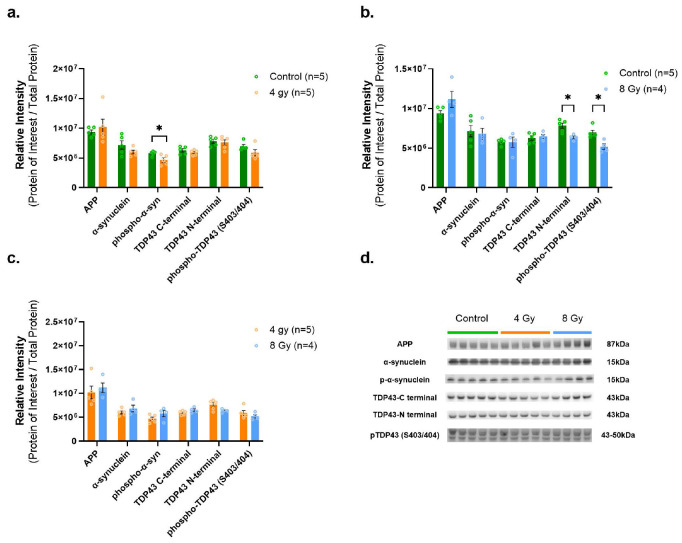
WB assessment of protein abundance levels of canonical neurodegenerative markers after long-term recovery from irradiation. Our analysis of neurodegenerative related proteins revealed decreased phospho-α-synuclein after 4 Gy (**a.**) and decreased TDP43-N terminal and phospho-TDP43 (S403/404) after 8 Gy (**b.**), and all other neurodegenerative related proteins were unchanged (**a.-c.**). (**d.**) Representative western blots for APP, alpha-synuclein, phospho-alpha-synuclein, TDP43-C terminal, TDP43-N terminal and phospho-TDP43 (S403/404) in hippocampus. Each blot was run in triplicate with all protein signal intensities normalized to total protein content. The graphs show the average of 3 runs. * indicates p<0.05 as determined by unpaired 2-tailed t-test. Error bars represent the standard error of the mean (SEM). Original full-length blots and total protein stain for each are presented in Supplementary Figure F9.

Furthermore, we observed a significant decreased level of phosphorylated α-synuclein in the 4Gy vs. Control group (p = 0.024, df = 8) (Fig. 7a). Also, TDP-43 N-terminal and phosphorylated TDP-43 were significantly decreased in 8Gy vs. Controls with p = 0.0098 (TDP43 N-terminal) and 0.0080 (phosphoTDP43), df = 7, respectively (Fig. 7b). As a quick technical reminder, inconsistencies between WB and MS-based proteomic findings are possibly due to the mismatched epitope regions of each specific antibody (as commercially available) binding sites vs. the peptide sequence tags for TMT MS-based proteomic identification not targeted to measure specific phosphorylated variants of certain proteins. Full blot images for each antibody and total protein expression are available in Supplemental Figs. F7-F9).

### dPCR analysis of gene expression confirms downregulation of MAPT

**Figure 8:**
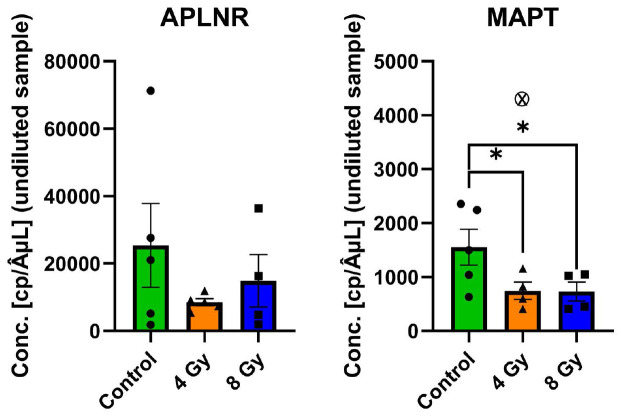
MAPT gene expression decreased in hippocampus after irradiation. dPCR quantification of mRNA expression identified no significant alteration in *APLNR* transcription, although variance was significantly different for 4 Gy exposure (a). *MAPT* was significantly decreased for both exposure levels (b) (4 Gy outlier indicated by X, excluded from analysis).

Of the targets chosen for WB verification, we further narrowed our selection down to *APLNR* and *MAPT* (Tau) (which we found to be one of the most surprising and meaningful findings in terms of possible radio-biological and neurodegeneration long-term effects) for further analysis using dPCR methods to measure their specific mRNA expression levels (Fig. 8). dPCR analyses showed that there were no significant changes in the activation of *APLNR* gene expression for either 4Gy or 8Gy vs. Control group. However, there was a significant difference in RNA expression variability between subjects in the 4Gy vs. 8Gy and Control group (Bartlett’s test for homogeneity of variance: Bartlett’s statistic (corrected) = 12.80; *p* = 0.0017). We did identify though a significant decrease in *MAPT* gene expression for both irradiation groups (4Gy and 8Gy) vs. Control (*p* < 0.05). dPCR-related graphs of the fluorescent intensities’ values are presented in **Supplemental Fig. F10**.

### Histopathological findings identified through IHC staining

**Figure 9:**
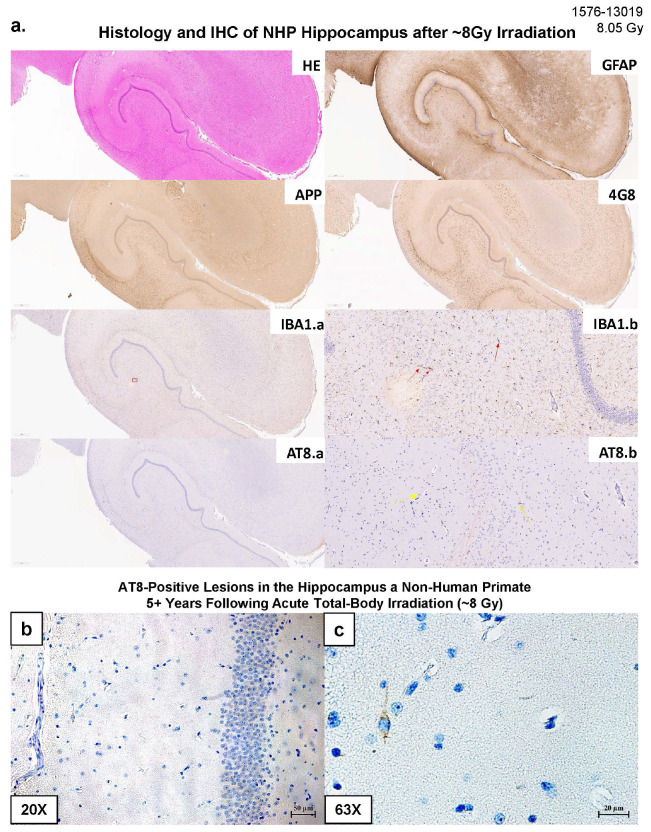
Histology and IHC of hippocampus after 8 Gy irradiation. Representative images of HE stains, GFAP, APP, 4G8, IBA1/AIF1, and AT8 in randomly selected case after 8 Gy exposure (a), with signs of IBA1 aggregation around lesions indicative of microglia reactivity. Representative images of immunohistochemical staining for hyperphosphorylated tau (AT8 antibody, brown) in the hippocampus of two different non-human primates, approximately 5-12 years after receiving 8 Gy of acute total-body irradiation. (b): an AT8-positive lesion in a 9 yrs-old animal; 20X magnification. (c) A higher magnification (63X-oil immersion) view of an intraneuronal neurofibrillary tangle from the same animal.

The systematic neuropathological assessment based on HE stain and GFAP and AIF1/IBA1 immunostaining of brain tissue sections including the hippocampus area, did not show overt signs of vascular, astroglial response, or diffuse microglial reactivity. The immunohistochemistry assessment for tau histopathology using the AT8 antibody (detecting insoluble tau protein hyperphosphorylated at serine 202 and threonine 205) did not show any pTau-positive lesions in the Control and 4Gy group (**Supplemental Fig. F6**). Interestingly though, two out of the four examined brains exposed to 8Gy irradiation showed isolated AT8-positive hippocampal lesions looking like pTau-positive pre/neurofibrillary tangles (NFTs) (Fig. 9). These two brains belonged to a 9- and 15-year-old subjects respectively, that is, they were much younger than those aged rhesus macaques in which AD-like tau-pathology has been previously reported (see discussion). Differences between these IHC-based findings and other molecular findings of this study may be related to the soluble vs. insoluble tau detectability of each specific technique used.

AIF1/IBA1 immunostaining identified, in the hippocampus, two cases of sparse microglial reactivity following 8Gy exposure (Fig. 9a), indicating a possible higher degree of microglial activation in comparison to the 4Gy and Control group (**Supplemental Fig. F6**; see discussion).

Finally, APP and 4G8 immunostaining did not reveal any signs of pathological abnormalities, such as diffuse axonal injury or extraneuronal amyloid deposits, following 4Gy or 8Gy exposure at this long-term recovery time point post-radiation (Fig. 9, **Supplemental Fig. F6**).

## Discussion

This study characterized the long-term effects of a single, total-body γ-radiation exposure on the adult non-human primate (NHP) hippocampus. Using a multi-modal approach combining mass spectrometry (MS)-based proteomics with WB, dPCR, and neuropathological validation, we addressed a significant gap in our understanding of the chronic neuro-molecular consequences of irradiation in a translationally relevant model. Our central finding from this small sample size is that radiation induces complex, non-linear, and dose-specific proteomic changes that persist for years after exposure. These results challenge simpler linear-threshold models of radiation damage and reveal distinct biological reprogramming events that have significant implications for radiotherapy, neurodegeneration, and brain aging.

A critical finding of this study is the non-linear molecular response of the hippocampus to radiation. Our proteomic analysis revealed a clear separation between irradiated and control groups, but more importantly, the 4Gy and 8Gy cohorts diverged significantly from each other, often in opposite directions (**Supplemental Figure F1**). This contrasts sharply with the widely accepted linear no-threshold (LNT) model, which posits that harm is directly proportional to the radiation dose^20^. Instead, our findings suggest that different radiation doses trigger qualitatively distinct neurobiological pathways rather than a simple, graded escalation of damage. This conclusion is further supported by the limited overlap in differentially abundant proteins between the two dose groups (**Supplemental Fig. F2**), indicating that the molecular signature of a 4Gy exposure is not merely a less severe version of an 8Gy exposure. This non-linear dose-response relationship has profound implications. For clinical radiotherapy, it suggests that minimizing dose to healthy tissue like the hippocampus does not just reduce the amount of damage but fundamentally changes its nature. The specific pathways disrupted at lower doses may be directly relevant to cognitive side effects, a point we explore below.

To better understand the functional consequences of these divergent proteomic profiles, we performed Gene Ontology (GO) and protein-protein interaction analyses (Fig. 3, **Supplemental Figs. F3-F5, Supplemental Table 2**). Though speculative, the results painted a clear picture of dose-specific functional impairment. In the 4Gy group, a sublethal dose for most NHPs, we observed significant enrichment of processes related to synaptic function, including glutamate receptor activity, calcium signaling, and synaptic structure (Fig. 3a, **Supplemental Fig. F4a, Supplemental Table T2**). This suggests that lower-dose radiation primarily dysregulates the molecular machinery of synaptic plasticity and neuronal communication^21-22^. Such a targeted disruption of synaptic integrity provides a direct biological mechanism for the long-term cognitive deficits observed in patients following radiotherapy, which may be driven less by overt cell death and more by a persistent state of synaptic dysregulation. This finding is consistent with previous work in a swine model exposed to lower doses of γ-radiation (1.79Gy), which also showed lasting effects on pathways related to neuronal function^5^.

In stark contrast, the 8Gy group showed enrichment in pathways related to catabolic processes, blood vessel development, chemotaxis, and oxygen sensing (Fig. 3b, **Supplemental Fig. F4b, Supplemental Table T2**). This proteomic signature points toward more global and damaging effects, including cellular degradation, vascular compromise, and sustained neuroinflammatory responses^23^. The direct comparison between the 8Gy and 4Gy groups further highlighted this divergence, with the higher dose uniquely impacting cytoskeletal organization and fibroblast proliferation (**Supplemental Fig. F4c, Supplemental Table T2**). This suggests that higher radiation doses may trigger extensive cellular restructuring and fibrotic responses, a known consequence of radiation damage in other tissues^24^.

Of the hundreds of proteins altered by radiation, only the Apelin Receptor (**APLNR**) was significantly affected across all comparisons, suggesting it may serve as a general, dose-independent biomarker of long-term radiation exposure in the hippocampus (Fig. 2). APLNR, a G-protein coupled receptor involved in neuroprotection and vascular regulation^25^, was consistently downregulated at the protein level. This novel result warrants dedicated investigation^26-28^, particularly because APLNR’s response to radiation is context-dependent. For example, studies have shown acute decreases in *APLNR* mRNA in the heart post-irradiation^29^, while APLNR silencing can enhance the radiosensitivity of prostate cancer^30^.

In our study, we examined a chronic timepoint (mean age of 6.7–9.4 years post-exposure) and found that the persistent decrease in APLNR protein may be a long-term consequence of an earlier, acute transcriptional downregulation. This has potential clinical relevance, especially in radiotherapy for brain tumors like glioblastoma, which are characterized by a pathological increase in Apelin/APLNR signaling^31-33^. Targeting the APLN/APLNR axis could potentially increase tumor sensitivity to radiation while mitigating damage to surrounding healthy tissue^30,34^. Our observation of decreased APLNR in normal primate brain tissue is consistent with radiation’s known impact on vascular morphology and suggests that chronic alterations in this pathway could contribute to radiation-induced ischemia and toxicity^35,36^, which can lead to cognitive decline years after exposure^37,38^.

Prompted by evidence of an accelerated aging phenotype in this NHP cohort^39^, and in light of recent identification of AD-like pathology in aging rhesus macaques^40^ and successful (and highly translational) development of a tau-based model of AD in rhesus monkeys^41^, we investigated established markers of neurodegeneration. Surprisingly, our MS-based proteomic analysis found that most canonical markers for AD(APP), PD (α-synuclein), and neuroinflammation (GFAP, AIF1/IBA1) were unchanged. The sole exception was MAPT, which encodes the Tau protein, a multi-functional microtubule-associated protein in the neuron with multifaceted functions including but critically not limited to axonal stabilization^42^; its abundance was significantly increased, but exclusively in the 8 Gy group (Table 1, Fig. 4). The change in MAPT abundance was moderate (and admittedly below the ± 20% FC (log2FC = 0.26) threshold we used as guidance during the MS-based proteomic analysis for determining the final list of significantly altered proteins following 4 Gy or 8 Gy exposure). However, it is possible that proteomic identification of the general MAPT protein is obscuring or diluting the altered abundance of one or more of the various forms of Tau, which is known to have diverse physiological and pathological functions depending on which form of tau and related interactions are driving the change^43^. The identification in this current study of isolated overall increase in Tau, without concurrent changes in other markers, suggests that a single high-dose radiation exposure may be sufficient to stress the cellular machinery for Tau homeostasis, potentially “priming” the brain for a future tauopathy. Our WB validation not only confirmed key proteomic findings (e.g., APLNR, CAMK2D) and null findings (e.g., GFAP, AIF1/IBA1), but also revealed subtle post-translational changes missed by MS. Specifically, we observed a decrease in phosphorylated α-synuclein after 4Gy exposure (Fig. 7a) and a decrease in N-terminal and phosphorylated TDP-43 after 8Gy exposure (Fig. 7b). The latter finding suggests an impact on normal TDP-43 function without inducing the toxic fragmentation characteristic of ALS/FTLD (as inferred by no change in C-terminal TDP-43; Fig. 7b)^44^.

The most striking result of our study emerged when we compared protein and mRNA levels for Tau. While WB analysis showed significantly higher levels of phosphorylated Tau (pTau181) in the 8Gy hippocampus compared to 4 Gy (Fig. 6c), dPCR quantification revealed that MAPT mRNA levels were, paradoxically, decreased (Fig. 8). This inverse relationship strongly indicates that post-translational regulation and protein stability, rather than ongoing gene transcription, dominate the control of pTau181 abundance years after irradiation. We hypothesize that the cellular machinery for protein degradation - namely the ubiquitin-proteasome and lysosomal pathways, which can be compromised by radiation^45,46^ - is failing to clear pTau181. Phosphorylation at the threonine-181 site can stabilize the Tau protein, making it less susceptible to clearance and causing it to accumulate^47^. This accumulation of pTau181 is a central event in the formation of neurofibrillary tangles (NFTs), a key pathological hallmark of AD^48,49^, and it has been well established that the neurodegenerative qualities of tau are highly specific to phosphorylation at discrete residue sites^50^. This finding offers a critical insight: the long-term neurobiological effect of high-dose radiation may be a chronic failure of protein clearance, creating a pathogenic state even after the initial transcriptional response has ceased. This is further supported by the divergent regulation of another phosphorylation site, pTau217, which decreased after 4Gy (Fig. 6a) and was unchanged at 8Gy relative to controls, highlighting the exquisite specificity of dose-dependent post-translational effects. Subcellular localization of tau and its distribution across different regions have huge implications for the unique functional roles of the many different varieties of tau^43^, and determining the specific locations of these altered tau proteins is necessary to elucidate the impact of radiation on tau-mediated mechanisms. It is also crucial that we replicate these measurements in other brain regions; assembly of tau into aberrant filaments driving neurodegeneration and dementia likely begins in a single brain area before spreading, and understanding the nature of tau throughout the brain (and the distribution of different filament structures) after long-term radiation recovery is key to determining whether the observed changes are protective or maladaptive^51^.

While previous work in this and other irradiated NHP cohorts has documented cerebrovascular remodeling, neuroinflammation, and white matter injury in other brain regions^52-55^, our study found limited evidence of such pathologies in the hippocampus. For instance, we observed only sparse microglial reactivity in two 8Gy cases (Fig. 9) and no significant changes in key white matter proteins. This discrepancy is likely attributable to several factors, including dose (our study used much lower total-body doses than the 40Gy fractionated whole-brain exposures in some prior work), recovery time, and inherent regional differences. Also, the hippocampus may possess a unique resilience to certain forms of radiation damage. Indeed, previous histopathological and imaging studies in this NHP population noted fewer signs of necrosis, microbleeds, or white matter lesions in the temporal region compared to others^52,55,1^. Our finding of only a trend towards increased fibronectin (FN1) at 8Gy (p = 0.078) further supports a less pronounced vascular and fibrotic response in the hippocampus at these doses and time points compared to that observed in the cortex^54^. This regional specificity is a crucial finding that warrants further exploration, as it underscores that the brain does not respond to radiation as a homogenous unit.

Beyond the limitation of small sample size, which is typical though for NHP studies due to the complex nature of working with these animals, we acknowledge the limitations in this study

First, there were discrepancies between our proteomic and WB validation results for some of the selected targets (e.g., CHRM5, SST). These may arise from methodological differences where MS identifies proteins by peptide sequence, while WB relies on antibody binding to specific epitopes, which can be affected by post-translational modifications or conformational changes.

Second, the 8Gy cohort represents a unique group of “survivors” who were inherently resilient enough to withstand a lethal dose of radiation for many years afterwards. Therefore, their molecular signature may reflect not only the detrimental effects of 8Gy but also underlying compensatory mechanisms that contributed to their survival. For example, the lack of widespread neurodegenerative pathology in this group could be a mark of their unique biological resilience.

Third, we have contextualized our findings as related to hippocampal changes, we also noted that while the hippocampus shows specific molecular shifts, it also appears more resilient to vascular pathology compared to other brain regions previously studied in this cohort.

Globally, while this study provides significant molecular insights, future larger works are needed to correlate these molecular changes with functional outcomes, such as cognitive and behavioral assessments. However, the novel pathways and targets identified here, particularly APLNR and the mechanisms governing Tau homeostasis, are of special relevance and they should be explored further as possible targets for therapeutic countermeasures to mitigate the long-term neurological consequences of radiation exposure and related cognitive decline.

With that in mind, it would be remiss not to explore the implications of these proteomic findings as they relate to cognitive functioning in the rhesus macaque. Although cognitive testing is not available for the specific subjects used in this study, there have been assessments performed on other NHPs within the Wake Forest University Radiation Late Effects Cohort (RLEC), namely, identification of an impairment in cognitive flexibility evident through a simple visual discrimination with reversal task^56^. The deficits were apparent at 7.2–8.3 years post irradiation, following a single acute exposure to 6.75–8.05 Gy gamma-irradiation in male monkeys aged 3.1–4.3 years old at the time of irradiation. Cognitive flexibility and reversal learning is primarily associated with the prefrontal cortex (although activation of diverse network connectivity extends to other regions like the premotor, orbitofrontal, parietal, temporal and occipital cortices), where studies have linked performance to several genes/proteins involved in dopaminergic and cholinergic systems (CHRNA4, COMT, DBH, DRD2, DRD4), serotonergic systems (HTR2A, HTR2C), and neuronal plasticity (PPP3R1/calcineurins and SYN3/synapsins)^57-61^. However, hippocampal mediated long term memory encoding and maintenance of working memory have recently been implicated in enhancing cognitive flexibility^62^. Specifically following 8 Gy exposure, our hippocampal data does show a significant increase in SYN3 (Log_2_(FC) = 0.34; *p* = 0.02), a significant decrease in PPP3R1 (Log_2_(FC) = −0.25; *p* = 0.01), and a trend towards increased HTR2A (Log_2_(FC) = 0.20; *p* = 0.09). Interestingly, variants of PPP3R1 and MAPT are implicated in functional decline in AD ^63^, while genetic deletion of astrocytic PPP3R1 in a mouse model of ADprevents cognitive decline and development of neuropathology^64^. Also, a recent study determined that a SYN3 knockout resulted in reduced cognitive flexibility^61^. Taken together, these cognitive and proteomic findings in ~ 8Gy exposed NHPs at long-term recovery timepoints converge on the possible conclusion that these alterations in the 8 Gy surviving group may be indicative of compensatory or even neuroprotective mechanisms.

In conclusion, our study demonstrates that a single radiation exposure initiates a complex, non-linear, and enduring reprogramming of the primate brain proteome. By revealing dose-specific pathological pathways - from synaptic dysregulation at lower doses to a failure of protein clearance at higher doses - these findings deepen our understanding of radiation-induced brain injury and provide a critical foundation for developing strategies to protect brain health in radiotherapy patients, astronauts, and other at-risk populations. More studies are needed to fully understand the complex biological processes driving long-term effects of radiation induced brain injury.

## Supplementary Material

This is a list of supplementary files associated with this preprint. Click to download.

- Table1editable.docx
- SupplementalInformation.pdf
- SupplementalTableT2DAVIDGOfunctionalenrichments.xlsx

## Figures and Tables

**Table 1:** Neurodegeneration Associated Proteins Identified in MS-based Proteomics Data

	4 Gy vs Control		8 Gy vs Control		8 Gy vs 4 Gy	
	*p* value	Log_2_ Fold Change	*p* value	Log_2_ Fold Change	*p* value	Log_2_ Fold Change
**APP**	0.161	0.07	0.073	0.13	0.824	0.06
**MAPT**	0.103	0.09	**0.025**	0.10	0.610	0.01
**SNCA**	0.949	0.02	0.401	0.15	0.272	0.13
**TARDBP**	0.921	0.08	0.552	−0.05	0.359	−0.13
**GFAP**	0.249	−0.20	0.283	−0.18	1	0.02
**AIF1**	0.966	−0.03	0.968	−0.07	0.881	−0.04

## Abbreviations

AIF1: allograft inflammatory factor 1 (also known as IBA1)
APLNR: apelin receptor
APP: amyloid precursor protein
CAMK2D: delta subunit of calcium/calmodulin-dependent protein kinase II
CHRM5: muscarinic acetylcholine receptor M5
dPCR: digital polymerase chain reaction
GFAP: glial fibrillary acidic protein
IBA1: ionized calcium-binding adapter molecular 1 (also known as AIF1)
IHC: immunohistochemistry
MAPT: microtubule-associated protein tau
MS: mass spectrometry
NHP: non-human primates
PPP3R1: calcineurin subunit B type 1
RIBI: radiation induced brain injury
RLEC: radiation Late Effects Cohort
SNCA: alpha-synuclein
SST: somatostatin
SYN3: synapsin III
TARDBP: TAR DNA Binding Protein (gene encoding TDP-43)
TDP-43: TAR DNA Binding Protein 43
WB: Western blot
